# Supplementary material for: A fault-intrusion-tolerant system and deadline-aware algorithm for scheduling scientific workflow in the cloud
Source: PeerJ Comput Sci. 2021 Nov 2;7:e747. doi: 10.7717/peerj-cs.747 (PMC8576554; doi:10.7717/peerj-cs.747)
Supplement: Supplemental Information 6 [file peerj-cs-07-747-s006.zip › Myproject1.1/docs/org/workflowsim/examples/WorkflowSimExample1.html]

WorkflowSimExample1


JavaScript is disabled on your browser.


- Overview
- Package
- Class
- Use
- Tree
- Deprecated
- Index
- Help

- Prev Class
- Next Class

- Frames
- No Frames

- All Classes

- Summary:
- Nested |
- Field |
- Constr |
- Method

- Detail:
- Field |
- Constr |
- Method


org.workflowsim.examples

## Class WorkflowSimExample1

- java.lang.Object
- - org.workflowsim.examples.WorkflowSimExample1

- ---

    

  ```
  public class WorkflowSimExample1
  extends java.lang.Object
  ```

  This WorkflowSimExample creates a workflow planner, a workflow engine, and
  one schedulers, one data centers and 20 vms. All the configuration of
  CloudSim is done in WorkflowSimExamplex.java All the configuration of
  WorkflowSim is done in the config.txt that must be specified in argument of
  this WorkflowSimExample. The argument should have at least: "-p
  path\_to\_config.txt"

  Since:
  :   WorkflowSim Toolkit 1.0

- - ### Constructor Summary

    Constructors

    Constructor and Description

    `WorkflowSimExample1()`
  - ### Method Summary

    Methods

    | Modifier and Type | Method and Description |
    | --- | --- |
    | `static void` | `main(java.lang.String[] args)` Creates main() to run this example This example has only one datacenter and one storage |

    - ### Methods inherited from class java.lang.Object

      `clone, equals, finalize, getClass, hashCode, notify, notifyAll, toString, wait, wait, wait`

- - ### Constructor Detail


    - #### WorkflowSimExample1

      ```
      public WorkflowSimExample1()
      ```
  - ### Method Detail


    - #### main

      ```
      public static void main(java.lang.String[] args)
      ```

      Creates main() to run this example
      This example has only one datacenter and one storage


- Overview
- Package
- Class
- Use
- Tree
- Deprecated
- Index
- Help

- Prev Class
- Next Class

- Frames
- No Frames

- All Classes

- Summary:
- Nested |
- Field |
- Constr |
- Method

- Detail:
- Field |
- Constr |
- Method
